# A Multimodal Diagnostic Model for Breast Cancer Invasiveness Based on Ultrasound Imaging and Serum Biomarkers

**DOI:** 10.3390/medicina61112010

**Published:** 2025-11-10

**Authors:** Dianhuan Tan, Yue Zhai, Zhengming Hu, Desheng Sun, Tingting Zheng

**Affiliations:** Shenzhen Key Laboratory for Drug Addiction and Medication Safety, Department of Ultrasound, Institute of Ultrasonic Medicine, Peking University Shenzhen Hospital, Shenzhen Peking University—The Hong Kong University of Science and Technology Medical Center, Shenzhen 518036, China; 2311210728@stu.pku.edu.cn (D.T.); 2311210741@stu.pku.edu.cn (Y.Z.);

**Keywords:** breast cancer invasiveness, multimodal diagnostics, ultrasound imaging, serum biomarkers, machine learning

## Abstract

*Background and Objectives:* Breast cancer invasiveness significantly impacts treatment strategies and prognosis. Combining ultrasound imaging modalities with serum biomarkers may improve diagnostic accuracy. We aimed to develop and validate a multimodal diagnostic model integrating ultrasound B-mode, Doppler imaging, and serum biomarkers for assessing breast cancer invasiveness. *Materials and Methods:* A multimodal diagnostic model was developed using ultrasound B-mode, Doppler imaging, and serum biomarkers from patients with invasive and non-invasive breast cancer. Machine learning algorithms, including Logistic Regression, Random Forest, and XGBoost, were applied to predict invasiveness, with performance evaluated using accuracy, precision, recall, F1-score, and AUC. *Results:* The multimodal model outperformed single-modality approaches, with XGBoost achieving the highest accuracy (88.90%) and AUC (0.930). The inclusion of specific serum biomarkers (e.g., CA125, CA15-3, CEA, and CA19-9) significantly enhanced diagnostic accuracy for breast cancer invasiveness. *Conclusions:* The proposed multimodal diagnostic model integrating ultrasound imaging and serum biomarkers provides a highly accurate and reliable method for assessing breast cancer invasiveness, offering potential to improve clinical decision-making and patient outcomes.

## 1. Introduction

Breast cancer remains one of the leading causes of cancer-related mortality among women globally, with tumor invasiveness serving as a critical determinant for guiding treatment strategies and predicting patient outcomes [1]. Traditionally, the malignancy of breast nodules is assessed using the BI-RADS classification system, which relies on imaging characteristics [2]. While effective at distinguishing benign from malignant nodules, this approach falls short in evaluating tumor invasiveness—a key factor in identifying high-risk patients. Accurate identification of invasive breast cancer is essential for the timely recognition of individuals at risk of tumor spread. Early detection in such cases allows for targeted and timely treatment interventions, ultimately improving patient outcomes and reducing mortality rates.

Pathological biopsy, considered the gold standard for assessing invasiveness, provides precise results but comes with significant limitations [3,4,5]. This invasive procedure can cause discomfort to patients and often requires multiple punctures, which increases the burden and risk for those undergoing evaluation [6,7]. Therefore, alternative methods that balance accuracy and patient convenience are essential.

Ultrasound imaging offers a non-invasive and accessible solution, particularly through the use of B-mode and Doppler modalities [8,9,10]. B-mode ultrasound captures morphological features of nodules, such as crab-like extensions and irregular edges, while Doppler ultrasound reflects blood flow patterns within the tumor, which can indicate vascular invasion [11]. These imaging characteristics suggest that ultrasound-based deep learning models hold significant potential for feature extraction and the non-invasive assessment of tumor invasiveness [12,13,14]. This underscores the importance of exploring advanced diagnostic approaches, such as integrating ultrasound imaging with deep learning techniques, to enhance the precision and efficiency of breast cancer management. However, the integration of these ultrasound features with serum biomarkers using machine learning to assess invasiveness specifically remains underexplored.

The advent of machine learning and multimodal diagnostic approaches presents transformative opportunities to integrate imaging data with molecular and clinical biomarkers [15,16,17]. Deep learning models have shown remarkable promise in medical imaging applications, such as tumor classification, treatment response prediction, and survival analysis. However, most existing studies are constrained to single modalities, focusing on either imaging or biomarkers, which limits their diagnostic comprehensiveness [18]. Notably, the use of multimodal machine learning models to evaluate breast cancer invasiveness remains underexplored. Ultrasound imaging, including B-mode and Doppler modalities, enhances the detection of morphological and vascular characteristics of breast tumors. Meanwhile, serum biomarkers provide a non-invasive avenue to complement imaging by capturing tumor biology at the molecular level. Nevertheless, relying solely on either approach can result in diagnostic uncertainties, highlighting the need for integrative strategies that combine their respective strengths.

To address these limitations, this study aims to develop and validate a novel diagnostic model that integrates ultrasound imaging (B-mode and Doppler) with serum biomarkers. By leveraging advanced machine learning algorithms, this approach seeks to enhance the accuracy and reliability of breast cancer invasiveness predictions. Using a large dataset and robust statistical validation, we aim to provide a comprehensive framework for combining imaging and biochemical data to improve clinical decision-making. This study contributes to bridging the gap between traditional diagnostic methods and advanced multimodal approaches in breast cancer management.

The integration of multimodal data, combining imaging techniques with biochemical markers, holds great promise for enhancing diagnostic accuracy [19,20]. Machine learning algorithms further enable the development of predictive models by identifying complex patterns in multimodal datasets [21]. This study aimed to develop and validate a multimodal diagnostic model that leverages advanced machine learning to integrate ultrasound imaging features (B-mode and Doppler) with serum biomarkers, with the goal of improving the assessment of breast cancer invasiveness, diagnostic precision, and clinical decision-making.

## 2. Materials and Methods

### 2.1. Study Population

**Figure 1:**
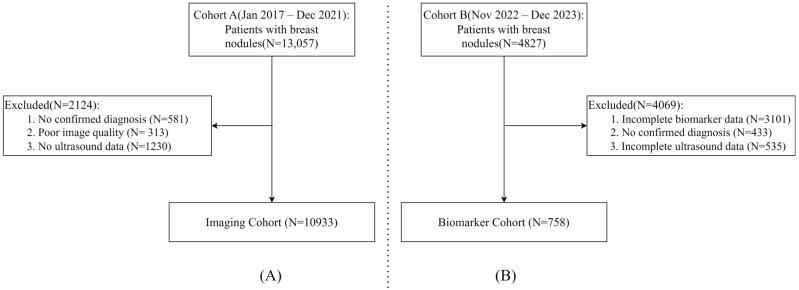
(**A**) Screening process for the imaging cohort. (**B**) Selection process for the biomarker cohort.

As shown in Figure 1, this study utilized two distinct cohorts to develop and evaluate the multimodal diagnostic model. As shown in Figure 1A, Cohort A consisted of breast cancer examinations conducted between 1 January 2017, and 31 December 2021. The inclusion of patients was based on the availability of a definitive pathological diagnosis. This diagnosis was established by pathological examination of tissue samples obtained via core needle biopsy, fine-needle aspiration, intraoperative frozen section, or postoperative surgical specimen, and served as the reference standard for determining cancer invasiveness. The exclusion criteria were as follows: (1) examinations without such a definitive pathological diagnosis, (2) poor image quality, and (3) missing either B-mode or Doppler ultrasound data.

As shown in Figure 1B, Cohort B consisted of patients with confirmed pathological diagnoses who underwent biomarker examinations between 1 November 2022, and 31 December 2023. The exclusion criteria were as follows: (1) incomplete biomarker data; (2) uncertain or provisional diagnoses; and (3) missing B-Mode or Doppler ultrasound data. There was no overlap between Cohort A and Cohort B. These cohorts provided the basis for constructing and validating the multimodal diagnostic model by integrating imaging data (ultrasound B-mode and Doppler) with serum biomarker information. The inclusion and exclusion processes are summarized in Figure 1, which illustrates the screening and selection steps for both cohorts.

### 2.2. Data Collection

Ultrasound images and clinical data were collected from patients included in Cohort A and Cohort B. All ultrasound examinations were performed using both B-mode and Doppler modalities to capture the morphological and vascular characteristics of the breast nodules.

The reference standard for classifying tumor invasiveness was the histopathological diagnosis obtained from core needle biopsy or surgical specimen examination. According to the established pathological criteria, invasive breast cancer was defined by the presence of malignant cells invading through the basement membrane into the surrounding stroma. In contrast, non-invasive breast cancer was characterized by the proliferation of malignant cells confined within the ductal-lobular system without stromal invasion [3].

**Figure 2:**
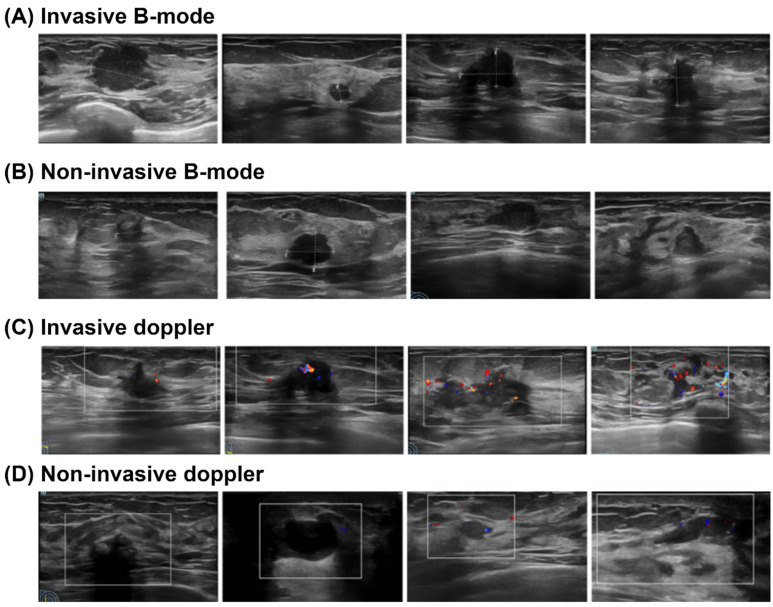
Representative ultrasound images of breast nodules with different pathological outcomes.

Representative ultrasound images illustrating the key diagnostic features that differentiate non-invasive from invasive breast cancer are provided in Figure 2A–D. Specifically, the B-mode image of a non-invasive cancer (Figure 2A) typically shows a hypoechoic mass with a regular shape and circumscribed margins, lacking spiculation or angularity, while its corresponding Doppler image (Figure 2B) shows sparse or absent vascular signals. In contrast, the invasive cancer on B-mode (Figure 2C) is characterized by an irregular mass with indistinct and spiculated margins, often accompanied by posterior acoustic shadowing. Conversely, the Doppler image of an invasive cancer (Figure 2D) reveals markedly increased vascularity with prominent penetrating or peripheral vessels, exhibiting rich blood flow signals often associated with a high resistive index (RI), which is indicative of active tumor angiogenesis. These critical morphological and vascular distinctions serve as the foundational visual cues for clinicians to preoperatively differentiate between invasive and non-invasive breast cancers.

For each patient, imaging data were matched with corresponding pathological and biomarker results. Pathological outcomes were used to classify nodules as invasive or non-invasive. Biomarker data were collected for patients in Cohort B and integrated into the diagnostic model. The dataset was curated to ensure consistency, with quality control applied to exclude incomplete or low-quality images and unmatched cases. These steps ensured a comprehensive and reliable dataset for model development and evaluation.

### 2.3. Serum Biomarker Assays

Serum biomarkers were analyzed for patients in Cohort B. The following biomarkers were measured using electrochemiluminescence immunoassays on an Alinity analyzer (Abbott Laboratories, Chicago, IL, USA) and a DXI800 analyzer (Beckman Coulter Life Sciences, Brea, CA, USA): cancer antigen 125 (CA125), cancer antigen 15-3 (CA15-3), carcinoembryonic antigen (CEA), cancer antigen 19-9 (CA19-9), alpha-fetoprotein (AFP), follicle-stimulating hormone (FSH), luteinizing hormone (LH), prolactin (PRL), progesterone (P), testosterone (T), and estradiol (E2). The assays were performed according to the manufacturer’s instructions. The normal reference ranges used in our clinical laboratory were as follows: CA125, <35 U/mL; CA15-3, <25 U/mL; CEA, <5 ng/mL for non-smokers; CA19-9, <27 U/mL; AFP, <7 ng/mL; FSH, 1.5–12.4 mIU/mL; LH, 1.7–8.6 mIU/mL; PRL, 4.79–23.3 ng/mL; P, 0.2–1.5 ng/mL; T, 0.29–1.67 nmol/L; E2, 99.1–447.8 pg/mL. These biomarkers were not evaluated in all patients; their assessment was specifically confined to patients in Cohort B, as detailed in Section 2.1.

### 2.4. Model Development

**Figure 3:**
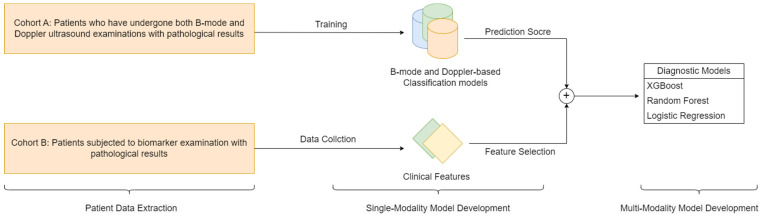
Workflow of multimodal diagnostic model development.

The development of the diagnostic model followed a multimodal approach based on a stacking ensemble framework, as outlined in Figure 3. This strategy was specifically designed to leverage the strengths of two distinct cohorts while rigorously preventing data leakage. The process encompassed three sequential stages: unimodal model development, prediction score generation, and final multimodal integration via a meta-learner.

To establish a robust foundation, separate unimodal models were developed parallel to their respective cohorts:

1. Imaging Model: Deep learning models (including EfficientNet, ResNet101, and ViT) were developed and validated exclusively on Cohort A, utilizing the B-mode and Doppler ultrasound images. A weighted loss function was employed during training to mitigate the effects of class imbalance. This approach assigned higher penalties for misclassifying minority (invasive) class samples, thereby compelling the models to pay closer attention to these critical, less frequent instances.
2. Biomarker Model: A separate predictive model was developed using the structured clinical biomarker data from Cohort B. Given the nature of tabular data, we employed XGBoost, which is well-suited for robust performance on such tasks. Feature selection was performed to identify the most informative serum markers for integration. This process involved first filtering biomarkers based on a univariate statistical significance threshold (*p* < 0.05). We then assessed multicollinearity among the significant features using the Variance Inflation Factor (VIF). All biomarkers retained for the final model had a VIF of less than 5, indicating acceptable levels of collinearity.

Following their development, these finalized unimodal models were used as feature extractors to generate prediction scores (probabilities of invasiveness) for their respective data types.

The core of our multimodal integration was a stacking ensemble method, which avoided combining the raw data from Cohorts A and B. Instead, we constructed a new dataset where each case was defined by two predictive features: the imaging model score and the biomarker model score. This integrated dataset was used to train a meta-learner (the XGBoost model) to optimally fuse these predictive streams. To ensure robustness against class imbalance at this stage, the XGBoost meta-learner was configured with appropriate class weighting. Crucially, the entire pipeline—from unimodal training to meta-learner optimization—was evaluated using a nested cross-validation strategy.

### 2.5. Statistical Analysis

The training environment utilized a GPU server equipped with Intel Xeon Platinum 8358 CPU and two NVIDIA RTX 4090. The software configuration included Ubuntu 18.04, PyTorch 2.1.2, Python 3.11, and MMPretrain 1.2.0. The dataset, containing two classes (“Benign” and “Malignant”), was formatted specifically for classification tasks.

For model-specific training configurations, EfficientNet used a batch size of 16 per GPU, ResNet101 employed a batch size of 16, and ViT was trained with a batch size of 8. The learning rate was adjusted proportionally for each model based on its batch size to ensure stable and effective optimization. Training was conducted over a maximum of 100 epochs.

The models were trained using different optimization strategies tailored to their architectures: EfficientNet and ResNet101 employed the SGD optimizer with momentum, while ViT utilized the AdamW optimizer. A Cosine Annealing learning rate scheduler was applied across all models to dynamically adjust the learning rate during training.

To ensure patient-level independence and prevent data leakage, the dataset was split at the patient level. Specifically, for both B-mode and Doppler ultrasound data, patients were first divided into training, validation, and test sets in a 7:1:2 ratio. All images belonging to a single patient were consistently assigned to the same subset (training, validation, or test).

During training, advanced techniques such as exponential moving average and synchronized normalization were incorporated. The model with the highest F1-Score on the validation set was selected and its best-performing checkpoint was automatically saved. This best model was then used for final evaluation on the independent test set, ensuring a robust and unbiased performance assessment. Model evaluation was conducted using a comprehensive suite of classification-specific metrics, including accuracy, precision, recall, F1-score, and Area Under the Receiver Operating Characteristic Curve (AUC), to provide a thorough analysis of performance throughout the training and testing process. Particularly in the context of class imbalance, the F1-score provides a more robust and informative measure than accuracy alone, as it balances the critical clinical priorities of identifying true positives (recall) and minimizing false alarms (precision). The observed dynamics between accuracy and F1-score in some of our single-modality models underscore the importance of selecting an evaluation metric that aligns with the clinical task—where correctly identifying invasive cases (high recall) is paramount, even at the potential cost of a slightly lower accuracy.

Clinical features were initially selected based on their statistical significance (*p* < 0.05) when comparing invasive and non-invasive breast cancer groups. Significant variables were identified as potential predictors of invasiveness. These features were incorporated into the model development to enhance diagnostic accuracy and reliability, ensuring that the integrated model was informed by clinically relevant factors.

For the ultrasound modalities, single-modality models were developed using B-mode and Doppler ultrasound data. These models were evaluated using key performance metrics, including accuracy, precision, recall, F1-score, and the area under the AUC. Receiver Operating Characteristic (ROC) curves were plotted for both B-mode and Doppler ultrasound models to visualize their classification performance and diagnostic potential. This step allowed for a detailed assessment of each modality’s ability to distinguish between invasive and non-invasive cases.

In constructing the multimodal diagnostic model, the output scores of the single-modality models for B-mode and Doppler ultrasound were first combined to create integrated models. Subsequently, clinical features with significant *p*-values were added to further enhance the models. The combined multimodal model was then evaluated and compared to the imaging-only model using the same performance metrics: accuracy, precision, recall, F1-score, and AUC. The ROC curve for the multimodal model was plotted to demonstrate its superior diagnostic performance, reflecting the synergistic value of integrating imaging and clinical data for assessing breast cancer invasiveness.

## 3. Results

### 3.1. Baseline Characteristics

The baseline characteristics of the study population provide a comprehensive overview of the data distribution and clinical features in the two cohorts used for model development and evaluation.

Cohort A comprised 10,933 unique ultrasound examinations, including both B-mode and Doppler ultrasound. This cohort contained a total of 33,002 B-mode images (6602 invasive and 26,400 non-invasive) and 15,419 Doppler images (2967 invasive and 12,452 non-invasive). These images represented 8739 unique cases, with 1517 invasive and 7222 non-invasive cases in the B-mode dataset, and 1352 invasive and 6930 non-invasive cases in the Doppler dataset, as summarized in Table 1.

Cohort B consisted of patients with clinical biomarker data, with mean values for invasive and non-invasive cases summarized in Table 2. Among the biomarkers analyzed, CA125 (*p* = 0.03), CA15-3 (*p* = 0.041), CEA (*p* = 0.04), and CA19-9 (*p* = 0.03) showed statistically significant differences between the invasive and non-invasive groups.

Together, the imaging and biomarker data establish a solid foundation for the development of multimodal diagnostic models, leveraging the complementary strengths of ultrasound imaging and clinical biomarkers to improve the assessment of breast cancer invasiveness.

### 3.2. Diagnostic Performance of Single-Modality Ultrasound

**Figure 4:**
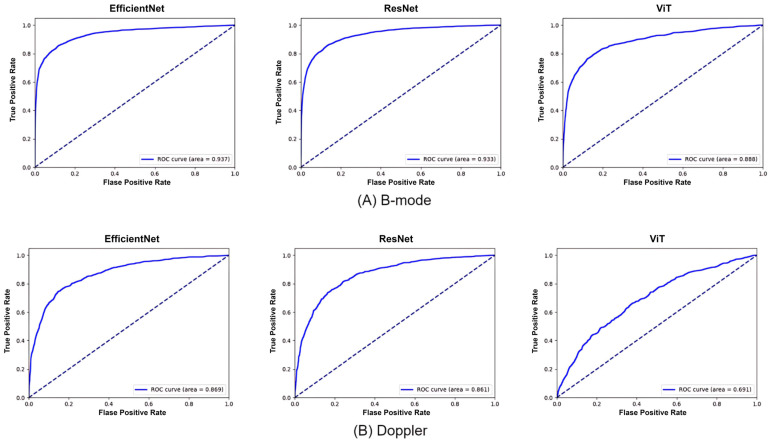
Receiver Operating Characteristic (ROC) curves of classification models. The dashed diagonal line represents the performance of a random classifier.

The diagnostic performance of single-modality models was evaluated separately for B-mode and Doppler ultrasound data, with key performance metrics summarized in Table 3 and Table 4. The ROC curves for these models are shown in Figure 4.

For B-mode ultrasound, EfficientNet achieved the highest overall performance, with an accuracy of 91.12%, an F1-score of 77.58%, and an AUC of 0.937. ResNet101 also demonstrated strong performance with an accuracy of 90.98%, an F1-score of 76.43%, and an AUC of 0.933. ViT, while slightly behind in performance, achieved an accuracy of 86.71%, an F1-score of 68.19%, and an AUC of 0.888. These results indicate that EfficientNet is particularly effective for leveraging B-mode ultrasound data in the classification of invasive versus non-invasive cases.

For Doppler ultrasound, EfficientNet achieved the highest performance with an accuracy of 86.13%, an F1-score of 63.39%, and an AUC of 0.869, followed by ResNet101 with an accuracy of 84.87%, an F1-score of 58.72%, and an AUC of 0.861. ViT showed comparatively lower performance, with an accuracy of 75.40%, an F1-score of 38.92%, and an AUC of 0.691. This performance gap may partially stem from the transformer-based architecture of ViT, which is less suited for capturing the localized blood flow features critical in Doppler ultrasound analysis.

### 3.3. Diagnostic Performance of Multimodal Techniques

The performance of multimodal diagnostic models integrating ultrasound features and clinical biomarkers was evaluated using Logistic Regression, Random Forest, and XGBoost. The results demonstrate a significant improvement in classification performance when using multimodal data compared to single-modality ultrasound models.

Table 5 summarizes the performance of models developed using ultrasound data alone, including both B-mode and Doppler scores. XGBoost achieved the highest F1-score (90.16%) and accuracy (87.50%), with an AUC of 0.894. Logistic Regression and Random Forest also showed strong performance, with F1-scores of 88.54% and 89.23%, respectively. However, the integration of multimodal data further enhanced the models’ predictive capabilities.

As shown in Table 6, the multimodal models outperformed their ultrasound-only counterparts across all metrics. XGBoost demonstrated the best overall performance, achieving an accuracy of 88.90%, an F1-score of 91.20%, and an AUC of 0.930. Logistic Regression and Random Forest also improved, with AUC values increasing to 0.917 and 0.918, respectively. These results indicate the added value of incorporating clinical biomarkers into the diagnostic framework, leading to more accurate and robust predictions.

**Figure 5:**
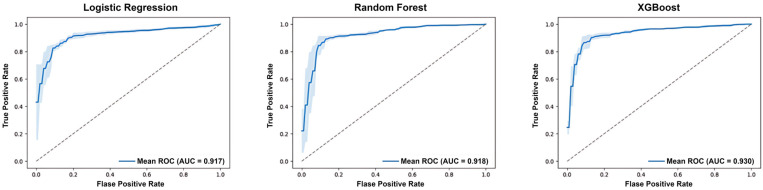
Receiver Operating Characteristic (ROC) curves of the multimodal diagnostic models. The dashed diagonal line represents the performance of a random classifier.

The ROC curves of the multimodal models, depicted in Figure 5, highlight the superior performance of XGBoost, which achieved the highest mean AUC. The shaded areas represent the variability in model performance, with all three models showing consistently strong discriminative ability.

**Figure 6:**
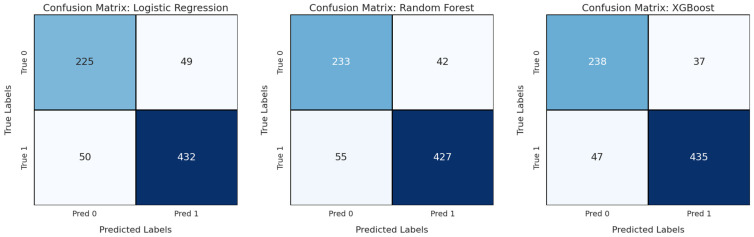
Confusion matrices for the multimodal diagnostic models. In the matrices, class 0 represents negative non-invasive cases, while class 1 represents positive invasive cases.

Confusion matrices for the multimodal models are presented in Figure 6, illustrating the distribution of true positive, true negative, false positive, and false negative predictions. Class 0 corresponds to negative non-invasive cases, while class 1 represents positive invasive cases. The matrices demonstrate the ability of XGBoost to achieve the most balanced classification, with fewer misclassifications compared to Logistic Regression and Random Forest.

Overall, these findings underscore the efficacy of multimodal approaches in leveraging both imaging and clinical data to enhance diagnostic performance, with XGBoost emerging as the most effective model in this study.

## 4. Discussion

### 4.1. Multimodal Advantage

The integration of imaging and biomarker data proved to be highly effective, as reflected in the superior performance of the multimodal models compared to ultrasound-only models. Among the algorithms tested, XGBoost consistently demonstrated the highest diagnostic performance, achieving an F1-score of 91.20% and an AUC of 0.930 using multimodal data. These improvements highlight the complementary strengths of ultrasound imaging and serum biomarkers.

Ultrasound provided structural and vascular information critical for differentiating invasive from non-invasive tumors, while biomarkers offered molecular-level insights that enhanced the model’s discriminative ability. Specifically, circulating biomarkers can offer crucial information for early diagnosis, risk assessment, and tracking disease progression at a molecular level, complementing imaging findings by reflecting the tumor’s biological aggressiveness and systemic impact [22,23]. This synergistic combination leads to a more comprehensive and accurate assessment of breast cancer invasiveness.

### 4.2. Performance of Single-Modality Models

**Figure 7:**
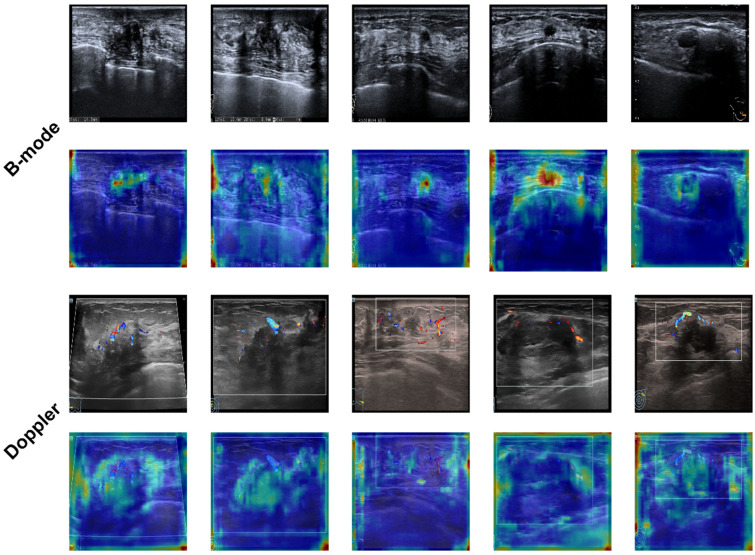
Examples illustrating model focus via heatmaps.

Analysis of single-modality models revealed that deep learning architectures could effectively extract features from ultrasound data for invasiveness assessment. Among the tested models, EfficientNet consistently outperformed ResNet101 and ViT in both B-mode and Doppler imaging, demonstrating its superior feature extraction capabilities. To enhance interpretability and provide insight into the features prioritized by our models, Grad-CAM visualizations were generated for the best-performing EfficientNet models across both modalities, as shown in Figure 7.

For B-mode ultrasound (Figure 7, rows 1–2), the Grad-CAM heatmaps (Row 2, overlaid on original B-mode images in Row 1) clearly indicate that the EfficientNet model primarily focused on the core lesion and its immediate morphological characteristics. Hotter regions (red/orange) in the heatmaps consistently highlight areas such as irregular margins, heterogeneous internal echotexture, and posterior acoustic features (e.g., posterior shadowing or enhancement). This observation strongly aligns with conventional ultrasound features used by expert radiologists for suspicious lesion evaluation and breast cancer invasiveness assessment, suggesting the model successfully learned clinically relevant patterns.

Conversely, Grad-CAM on Doppler images (Figure 7, rows 3–4; Row 4 overlaid on original Doppler in Row 3) primarily highlighted regions of increased vascularity and abnormal blood flow patterns within and around the lesions, identifiable by the concentrated red/orange activations where color flow signals are present. However, despite the model’s attention to vascular features, Doppler-based models generally exhibited comparatively lower performance than their B-mode counterparts. This reduced performance likely stems from the inherent complexity, variability, and often subtle nature of blood flow patterns in Doppler imaging, which can be challenging even for advanced deep learning architectures to robustly capture and interpret across diverse cases with consistent discriminative power, even when these features are highlighted by Grad-CAM. These interpretability analyses through Grad-CAM are thus indispensable for understanding model behavior and fostering trust in its clinical application, paving the way for future model refinements.

(A) B-mode ultrasound images and Grad-CAM heatmaps. Row 1 displays five representative original B-mode ultrasound images of breast lesions. Row 2 shows their corresponding Grad-CAM heatmaps, overlaid on the B-mode images. The heatmaps predominantly highlight the lesion core, its margins, and internal echotexture (e.g., irregular shape and heterogeneous texture strongly activated in Column 1 and 3), consistent with morphological features recognized by radiologists for malignancy assessment.

(B) Doppler ultrasound images and Grad-CAM heatmaps. Row 3 presents the corresponding original Doppler ultrasound images for the same five lesions, emphasizing vascularity (red/blue color flow). Row 4 displays Grad-CAM heatmaps overlaid on these Doppler images. Here, activations primarily focus on regions of increased vascularity or abnormal blood flow patterns (e.g., strong activations localized around conspicuous blood vessels in Column 2 and 4).

### 4.3. Comparative Analysis of Model Architectures

Our comparative analysis of different deep learning architectures revealed significant performance variations. Among the tested models, EfficientNet consistently outperformed both ResNet101 and the ViT across both ultrasound modalities. The relatively lower performance of ViT, particularly for Doppler data where it achieved an AUC of only 0.691, warrants further discussion. We hypothesize that this underperformance can be attributed to ViT’s global self-attention mechanism, which may be less effective at discerning precise, localized morphological and vascular abnormalities compared to the inherently local feature extraction of convolutional architectures like EfficientNet. This finding has an important clinical implication: it suggests that for the task of identifying invasive breast cancer from ultrasound images, capturing fine-grained, localized details (such as irregular margins or specific vascular patterns) is more critical than establishing long-range global dependencies across the entire image. This insight is valuable for guiding future model selection and development in this specific medical imaging domain.

### 4.4. Practical and Clinical Implications

The proposed multimodal model offers a non-invasive, accurate, and reliable tool for assessing breast cancer invasiveness, addressing limitations of conventional diagnostic methods. As our model comparison revealed (Section 4.3), convolutional networks are particularly well-suited for this task. While pathological biopsy remains the gold standard for definitive diagnosis, its invasiveness and associated risks limit its routine applicability for initial assessment and serial monitoring. By contrast, the integration of non-invasive ultrasound imaging with clinical biomarkers in a machine learning framework provides a practical alternative for early and accurate assessment, potentially enabling personalized treatment strategies and improved patient outcomes.

Recent advancements in deep learning applied to breast ultrasound have demonstrated significant potential in tackling various diagnostic challenges. For instance, studies have successfully leveraged ultrasound features to distinguish between benign and malignant breast lesions, and even to differentiate complex cases like mass mastitis from invasive breast cancer using deep learning radiomics nomograms [24]. Beyond general lesion characterization, sophisticated AI models applied to routine imaging data have shown promise in predicting more nuanced and critical indicators of tumor biological aggressiveness. Specifically, a deep learning radiomics nomogram based on B-mode and Doppler ultrasound images has been developed for the preoperative prediction of lymphovascular invasion (LVI) in invasive breast cancer, showcasing excellent performance and clinical utility [25]. Furthermore, similar efforts applying multiphase Dynamic Contrast-Enhanced Magnetic Resonance Imaging (DCE-MRI) radiomics have also successfully predicted LVI status, highlighting the value of non-invasive imaging in assessing this crucial prognostic factor across different modalities [26].

Building upon these progresses, our study extends the application of deep learning on ultrasound beyond benign-malignant differentiation to the broader and clinically critical task of assessing overall tumor invasiveness. By incorporating not only comprehensive ultrasound characteristics (B-mode and Doppler) but also essential circulating biomarkers, our model provides a more holistic, comprehensive, and accessible approach for clinicians to gain deeper insights into tumor biology early in the diagnostic pathway. This non-invasive assessment could guide decisions regarding the necessity and timing of biopsy, pre-surgical planning, and the selection of neoadjuvant therapies, ultimately contributing to more tailored and effective patient management.

### 4.5. Limitations and Future Directions

Despite its promising results, this study has several limitations. First, the data were collected retrospectively from a single center, which may limit the generalizability of the findings. Multicenter validation studies with diverse populations are needed to confirm the model’s robustness. Second, although the multimodal model demonstrated strong performance, the imbalance between invasive and non-invasive cases in the dataset could influence model training and evaluation. Future studies should explore more balanced datasets or employ advanced techniques for handling class imbalance.

Additionally, while the transformer-based ViT model underperformed in this study, hybrid architectures combining convolutional layers with transformer modules may enhance the extraction of localized features, particularly for Doppler data. Future work could also explore the inclusion of additional biomarkers or imaging modalities, such as contrast-enhanced ultrasound or MRI, to further improve diagnostic precision.

### 4.6. Conclusions

In summary, this study demonstrates the significant potential of a multimodal diagnostic approach that combines ultrasound imaging and clinical biomarkers for assessing breast cancer invasiveness. The results demonstrated that the multimodal approach significantly outperformed single-modality models, providing a robust and accurate method for classifying invasive and non-invasive breast cancer cases. The findings emphasize the importance of integrating complementary data sources to achieve higher diagnostic accuracy and reliability. The proposed model, particularly the XGBoost-based framework, provides a promising avenue for improving clinical decision-making and patient outcomes. Further research should focus on validating and refining the model for broader clinical applications, paving the way for its adoption in routine breast cancer management.

## Figures and Tables

**Table 1:** Data distribution of the cohort A.

Ultrasound Modality	Invasive Images	Non-Invasive Images	Invasive Cases	Non-Invasive Cases
B-mode	6602	26,400	1517	7222
Doppler	2967	12,452	1352	6930

**Table 2:** Clinical characteristics of cohort B. All values represent mean serum concentrations. CA125, CA15-3, and CA19-9 are reported in U/mL; CEA and AFP in ng/mL; FSH and LH in mIU/mL; PRL in ng/mL; Progesterone (P) in ng/mL; Testosterone (T) in nmol/L; and Estradiol (E2) in pg/mL.

Marker	Invasive Mean	Non-Invasive Mean	*p*-Value	Significant
CA125	26.10	23.31	0.03	Yes
CA15-3	17.28	17.41	0.041	Yes
CEA	11.62	5.46	0.04	Yes
CA19-9	67.99	34.71	0.03	Yes
AFP	2.44	2.55	0.73	No
FSH	29.69	32.92	0.542	No
LH	18.00	17.68	0.41	No
PRL	22.11	20.85	0.371	No
P	2.67	1.82	0.796	No
T	0.37	0.34	0.84	No
E2	62.08	62.50	0.383	No

**Table 3:** Performance metrics of various models on the B-mode test set.

Model	Accuracy (%)	Precision (%)	Recall (%)	F1-Score (%)	AUC
ResNet101	90.98	79.85	73.29	76.43	0.933
ViT	86.71	65.27	71.39	68.19	0.888
EfficientNet	91.12	78.15	77.02	77.58	0.937

**Table 4:** Performance metrics of various models on the Doppler test set.

Model	Accuracy (%)	Precision (%)	Recall (%)	F1-Score (%)	AUC
ResNet101	84.87	61.47	56.21	58.72	0.861
ViT	75.40	37.08	40.94	38.92	0.691
EfficientNet	86.13	64.04	62.75	63.39	0.869

**Table 5:** Classification performance of three diagnosis models built using imaging-derived prediction scores.

Model	Accuracy (%)	Precision (%)	Recall (%)	F1-Score (%)	AUC
Logistic Regression	85.53	89.47	87.63	88.54	0.909
Random Forest	86.09	87.88	90.62	89.23	0.898
XGBoost	87.50	90.62	89.69	90.16	0.894

**Table 6:** Classification performance of diagnosis models built using multimodal data.

Model	Accuracy (%)	Precision (%)	Recall (%)	F1-Score (%)	AUC
Logistic Regression	86.79	89.83	89.42	89.61	0.917
Random Forest	87.18	91.15	88.58	89.79	0.918
XGBoost	88.90	92.17	90.25	91.20	0.930

## Data Availability

The data used in this study contain sensitive patient information and are therefore not publicly available to protect patient privacy.
